# Measuring Morbidity Associated with Urinary Schistosomiasis: Assessing Levels of Excreted Urine Albumin and Urinary Tract Pathologies

**DOI:** 10.1371/journal.pntd.0000526

**Published:** 2009-10-06

**Authors:** José C. Sousa-Figueiredo, María-Gloria Basáñez, I. Simba Khamis, Amadou Garba, David Rollinson, J. Russell Stothard

**Affiliations:** 1 Wolfson Wellcome Biomedical Laboratories, Department of Zoology, Natural History Museum, London, United Kingdom; 2 Department of Infectious Disease Epidemiology, Imperial College London, London, United Kingdom; 3 Helminth Control Laboratory Unguja, Helminth Control Programme, Zanzibar, Tanzania; 4 Réseau International Schistosomoses Environnement Amenagements et Lutte (RISEAL-Niger), Niamey, Niger; Centre Suisse de Recherches Scientifiques, Infectious Diseases/Helminth Infections

## Abstract

**Background:**

Urinary schistosomiasis is responsible for a variety of debilitating conditions; foremost perhaps are urinary tract pathologies (UTPs). Although portable ultrasonography can be used to detect UTPs visually, there is still a need for rapid morbidity assessment (henceforth referred to as RaMA) tools that can be deployed in the field during implementation, monitoring and evaluation of control programmes. We therefore aimed to determine associations between excreted urine-albumin, as measured using a HemoCue photometer, and UTPs, as detected by ultrasonography, in children and adults from an urinary schistosomiasis endemic area in Zanzibar.

**Methodology/Principal Findings:**

In a survey of 140 school-children of both sexes (aged 9 to 15 yr) and 47 adult males (≥16 yr) on the island of Unguja, the prevalence of egg-patent urinary schistosomiasis was 36.4% (CI_95_ 28.5–45.0%) and 46.8% (CI_95_ 32.1–61.9%) (*P* = 0.14), and that of UTPs was 39.4% (CI_95_ 31.0–48.3%) and 64.4% (CI_95_ 48.8–78.1%) (*P* = 0.006), respectively. In school-children, raised urine-albumin concentrations (>40 mg/L) were associated, albeit non-significantly, with prevalence of infection (OR = 3.1, *P* = 0.070), but more specifically and significantly with the prevalence of micro-haematuria (OR = 76.7, *P*<0.0001). In adults, elevated urine-albumin excretion was associated with UTPs, particularly lesions of the bladder wall (OR = 8.4, *P* = 0.013). Albuminuria showed promising diagnostic performance, especially in school-aged children with sensitivity of 63.3% and specificity of 83.1% at detecting lower UTPs, i.e. bladder-wall lesions (ultrasonography as ‘gold standard’).

**Conclusion/Significance:**

This study indicates that albuminuria assays could be used as a RaMA tool for monitoring UTP prevalence during control programmes, as well as a tool for selecting those with more chronic bladder-wall lesions without resorting to ultrasonography.

## Author Summary

Urinary schistosomiasis is a debilitating disease caused by a parasitic worm that dwells in the blood vessels, particularly those surrounding the human bladder wall. Although not directly associated with high patient mortality, this disease is linked to both short-term morbidity, e.g. visible blood in urine (acute), as well as long-term sequelae, e.g. urinary tract pathologies (chronic). Numerous control programmes based upon chemotherapy have been implemented in sub-Saharan Africa in an attempt to reduce the burden of disease inflicted, particularly in children. Although there are rapid tests to assess the prevalence of acute manifestations of disease (i.e. blood in urine), namely urine-reagent strips, monitoring of chronic manifestations (i.e. urinary tract pathologies) is still rather laborious, time-consuming and requires specialised equipment, e.g. portable ultrasonography, as well as highly trained staff. This study has attempted to evaluate associations between albuminuria (albumin in urine, a new application for the HemoCue photometer) and urinary tract pathologies, and consequently assess this new biochemical marker as a potential rapid proxy of chronic disease sequelae typical in children in areas where urinary schistosomiasis is of public health importance.

## Introduction

Urinary schistosomiasis is caused by infection with the parasitic trematode *Schistosoma haematobium*. Disease-associated morbidities range from blood in urine (haematuria), both visible (macro-haematuria) and non-visible (micro-haematuria), to more chronic sequelae such as anaemia, urinary tract pathologies (UTPs) and developmental retardation [1],[2],[3]. Urinary schistosomiasis is considered under the umbrella of neglected tropical diseases (NTDs) [4]. There are several control initiatives working at national levels within endemic zones in sub-Saharan Africa administering regular distribution of praziquantel, the drug of choice to combat urinary schistosomiasis [5],[6]. One such example is the Zanzibar programme on Unguja Island for the reduction of urinary schistosomiasis and prevention of morbidity (‘*Piga Vita Kichocho*’ in Kiswahili), which since 2003 has provided treatment to more than 132,000 school-children yearly [7],[8],[9]. Monitoring of control programmes is crucial in order to determine their effectiveness, as well as to enable prompt identification of deficiencies and implementation of corrective or realignment measures [10]. For schistosomiasis control initiatives, this process involves assessing the temporal and spatial trends of infection prevalence and intensity in the presence of treatment, as well as that of morbidity indicators. The latter is typically conducted through the use of cost-effective and simple (rapid assessment) techniques, of which the most appropriate and commonly used for urinary schistosomiasis is the screening of individuals for macro- and micro-haematuria [2],[11],[12].

More recently, with the availability of the HemoCue photometer assay (HemoCue, Ängelholm, Sweden), raised levels of excreted albumin in urine (>40 mg/L) have been found indicative of active *S. haematobium* infections in school-children [7], building on previous observations [13],[14]. Whilst this technique may never substitute parasitological diagnosis nor urine-reagent strip tests, it could play an important additional role in the monitoring of control programmes, particularly due to the postulated relationship between urine-albumin excretion and UTPs [13], as well as their amelioration following treatment [1]. Although ultrasonography has many advantages (discussed in [15]) and is considered the ‘gold standard’ for diagnosing schistosomiasis-related UTPs [1],[16],[17], it is unfortunately time-consuming, and requires heavy equipment, on site electricity (or availability of a generator), and presence of trained staff [18], which somewhat limit its large-scale applicability in many endemic areas. Other shortcomings include low sensitivity to mild lesions and early inflammatory changes, as well as inter- and intra-observers' variation in scoring observed UTPs [18],[19]. Therefore, there is still a need for low-cost, high throughput, practical, reliable, and rapid morbidity assessment (henceforth referred to as RaMA) methods for the measuring and monitoring of schistosomiasis-induced UTPs.

The aims of the present study were to assess the association between excreted urine-albumin, a known proxy of *S. haematobium* infection in children, and UTPs, as detected by ultrasonography, in both school-aged children (9 to 15 year olds) and adult males (≥16 year olds). In addition, we sought to determine the prevalence of UTPs in rural areas of Unguja Island, Zanzibar, in the face of ongoing schistosomiasis control.

## Materials and Methods

### Ethical approval, informed consents and de-worming treatment

The Ministry of Health, Zanzibar, and Imperial College of Science, Technology and Medicine, London, granted ethical approval for this study (application no. ICREC 03.36). At each school, written or oral informed consents were given by the school head-teacher and pupils, respectively. In the health centre, the participants granted their written informed consent, which were documented using checklists. Individuals found to be positive for infection with *S. haematobium* were treated with a standard dose of praziquantel (40 mg/kg), and treatment was offered to anyone not examined in the study but who wished to receive it.

### Study area and population

The data were collected in May 2005 as part of the surveillance and monitoring of the second round of mass praziquantel treatment of the ‘*Piga Vita Kichocho*’ programme [9]. The survey took place in three primary schools on northern Unguja: Chaani, Kinyasini and Mwera, where a total of 140 children, between the ages of 9 and 15 years, were examined (with sample sizes of 60, 36, and 44 for each school, respectively). To investigate further the levels of morbidity within the general community, a small walk-in (random) cross-sectional survey of 47 adult males (≥16 years of age) took place at Chaani Health Centre. All four locations are inside the schistosomiasis-endemic area of Unguja, where the intermediate snail host, *Bulinus globusus*, persists [20]. Details of the location of the schools and health centre on Unguja have been presented elsewhere [8],[9].

### Interview questionnaire

Each individual was interviewed by a member of staff of the Helminth Control Laboratory, Unguja, and asked a suite of 20 questions, recording demographic information (age, sex), general self-reported health conditions (e.g. headache, abdominal and back pains, pain on urination) and other variables (e.g. access to drinking water and treatment, as well as bladder continence). Copies of the questionnaire are available upon request (corresponding author).

### Parasitological and morbidity survey

Urine dipstick tests were conducted, yielding the urine's chemical/physical profile: pH, specific gravity, protein content, glucose, blood (micro-haematuria), nitrite and ketone levels, as well as leukocyte presence (Multistix, Bayer, UK). Urine-reagent strips (Hemastix, Bayer, UK) were used as a cross-check for micro-haematuria prevalence [12]. Urine-albumin levels were measured using the Albumin-HemoCue photometer (HemoCue, Ängelholm, Sweden) [7]. (When comparing urine-albumin measurements obtained with the photometer against those obtained using the more general Multistix test, the former is protein-specific and provides a whole dilution range for finer accuracy, whereas the latter (the dipstick) saturates at lower albuminuria levels, yielding a coarser reading.) Macro-haematuria and urine's opacity (turbidity) were assessed visually (with the use of a barcode chart). From each sample of mid-morning urine, 10 ml were syringed through a Millipore filter (12 µm polycarbonate filter) for detection of *S. haematobium* eggs, and infections categorised as light (1–10 eggs), medium (11–49 eggs), or heavy (≥50 eggs) [7].

### Portable ultrasonography

Using a portable ultrasound machine (Aloka SSD-500, 3.5 MHz external probe, Aloka Inc., Japan), a variety of typical upper and lower urinary tract morbidities associated with urinary schistosomiasis (affecting organs such as kidneys, bladder, and ureters) were assessed and recorded in 132 school-children and 45 adult males, according to World Health Organization standardized examination protocols and severity scores [21], which are available at http://whqlibdoc.who.int/hq/1991/TDR_SCH_ULTRASON_91.3.pdf.

### Diagnostic tests

Micro- and macro-haematuria, visual opacity (turbidity) and raised urine-albumin concentrations (>40 mg/L) were tested qualitatively as alternative urinary schistosomiasis diagnostics, and for each, sensitivity (SS), specificity (SP), positive predictive value (PPV) and negative predictive value (NPV) were calculated against the ‘gold standard’ of parasitological diagnosis in urine filtrates. The same was done for urine-albumin assays as a potential rapid diagnostic test for UTPs against the ‘gold standard’ of ultrasonography. SS and SP indicate, respectively, the test's ability to identify correctly an individual as infected/diseased, or uninfected/healthy. PPV indicates the probability that in case of positive diagnosis, the patient is truly infected/diseased, whereas NPV indicates the probability that a negative diagnosis is in fact truly uninfected/healthy [22]. The diagnostic performance of each test was calculated using school-children and adults as separate target populations and (exact) 95% confidence intervals were calculated for the various point estimates of diagnostic precision [23] as described below.

### Statistical analysis

Data were collected from each individual using pre-format data sheets, which were then entered onto an electronic format using Microsoft Excel™. The data thus collated were analysed using MS Excel™ and R statistical package v 2.8.1 [24]. For prevalence values (and measures of diagnostic performance), 95% confidence intervals (CI_95_) were estimated using the exact method [25]. Prevalence comparisons were performed using (one-tailed) Fisher's exact modification of the 2×2 chi-squared test [26]. For infection intensity values, the geometric mean of Williams, GM_W_ [27] was chosen as the measure of central tendency due to the typical over-dispersion present in this type of data, and (asymmetric) CI_95_ values were estimated [28].

Univariate and multivariate analyses were carried out using logistic regression to assess for potential statistical associations between (parasitological) infection status, albuminuria (defined as a concentration >40 mg/L according to [7]), urine's chemical/physical profile, questionnaire data, and results of infection/morbidity diagnostic tests. A final, most parsimonious yet adequate model was chosen, defining infected individuals as cases and incorporating all variables from the questionnaire, using backwards stepwise selection and the Akaike information criterion (AIC). The AIC represents a balance between maximising the log-likelihood of the model and penalising it for a larger number of parameters; a model with a lower value of AIC is considered to be a better model [29]. Similar methodology was applied in identifying potential statistical associations between infection status and the different UTPs investigated (pathologies of the bladder, ureters, renal pelvis, and prevalence of calcifications). In parallel, a third model was established using stepwise regression, defining individuals with raised urine-albumin levels (>40 mg/L) as cases, in an attempt to identify explanatory variables associated with elevated albumin in the urine of school-children and adult males. Final models for infection status incorporated sex (in children) and age (children and adult males), even in the absence of significance at the univariate level, since they have previously been identified as risk factors for schistosomiasis [30],[31]. Similarly, school was included in the final model of infection to account for possible intra-correlation in the data, as children that go to the same school are more likely to share exposures/diet/genetics than children attending different schools. For each variable, adjusted odds ratio (OR) and *P*-values were calculated. A *P*-value<0.05 was considered indicative of statistical significance.

## Results

### Patient characteristics

The mean age of the school-children was 11.4 years (median = 11 years), and 60% of individuals were between the ages of 10 and 12, with a female to male ratio of 0.80. The mean age of the adult males from the Chaani Health Centre was 29.6 years (median = 24), and 71% were between 20 and 40 years of age. A total of 84% of school-children and 64% of adult males reported to have access to potable water. Anti-schistosomiasis treatment coverage was close to 75% in school-children (ranging from 58% at Chaani school to 80% at Kinyasini school), whereas only 40% of the adult males reported to have had treatment. A total of 35% of school-children and 62% of adult males reported to feel pain on urination, and 14% of school-children and 23% of adult males reported to wake up at night for urination.

### Urinary schistosomiasis and urinary tract pathologies

Urinary schistosomiasis was more prevalent (albeit not significantly, *P* = 0.14) in the adult males surveyed at the Health Centre [46.8%, GM_W_ = 2.84 (CI_95_ 2.36–3.31) eggs/10 ml of urine] than in the school-children [36.4%, GM_W_ = 2.17 (CI_95_ 1.90–2.44) eggs/10 ml of urine]. Data from Kinyasini school revealed a much higher *S. haematobium* infection prevalence [72.2%, GM_W_ = 10.61 (CI_95_ 10.05–11.17) egg/10 ml of urine] than in the remaining schools (*P*<0.0001) (Table 1). When categorised by intensity of infection, 19.2% of adult males and 15.7% of school-children (*P* = 0.37) were heavily infected by *S. haematobium* (≥50 eggs/10 ml of urine), accounting for 41.0% and 43.1% of all recorded infections in these age-groups, respectively. Of particular note again is Kinyasini School, where 41.7% of school-children were heavily infected by *S. haematobium* (in comparison with 6.7% in the remainder, *P*<0.0001), accounting for 57.7% of all recorded infection at this school (see Table 1, which includes the CI_95_ for the prevalence values).

**Table 1:** Prevalences values (and CI_95_) of *S. haematobium* infection (microscopy), micro-haematuria (urine-reagent strip), macro-haematuria (visual blood in urine), turbid urine (urine visual opacity), raised urine-albumin (HemoCue) and urinary tract pathologies (ultrasonography) in school-children enrolled in Chaani, Kinyasini, and Mwera schools, and adult males attending the Chaani Health Centre, Unguja, Zanzibar.

Condition	School-children, both sexes (9–15 yr)				Adult males (≥16 yr)
	Chaani (*N* = 60)	Kinyasini (*N* = 36)	Mwera (*N* = 44)	All schools (*N* = 140)	Health Centre (*N* = 47)
Urinary schistosomiasis (egg positive)	26.7 (16.1–39.7)	72.2 (54.8–85.8)	20.5 (9.8–35.3)	36.4 (28.5–45.0)	46.8 (32.1–61.9)
*Light* (1–10)	5.0 (1.0–13.9)	11.1 (3.1–26.1)	9.1 (2.5–21.7)	7.9 (4.0–13.6)	17.0 (7.7–30.8)
*Moderate* (11–49)	13.3 (5.9–24.6)	19.4 (8.2–36.0)	6.8 (1.4–18.7)	12.9 (7.8–19.6)	10.6 (3.6–23.1)
*Heavy* (≥50)	8.4 (2.8–18.4)	41.7 (25.5–59.2)	4.6 (0.6–15.5)	15.7 (10.1–22.8)	19.2 (9.2–33.3)
Micro-haematuria	33.3 (21.7–46.7)	83.3 (67.2–93.6)	18.2 (8.2–32.7)	41.4 (33.2–50.1)	59.6 (44.3–73.6)
Macro-haematuria	11.7 (4.8–22.6)	8.3 (1.8–22.5)	2.3 (0.1–12.2)	7.9 (4.0–13.6)	4.3 (0.5–14.5)
Turbid urine	38.3 (26.1–51.8)	63.9 (46.2–79.2)	18.2 (8.2–32.7)	38.6 (30.5–47.2)	51.1 (36.1–65.9)
Raised urine-albumin (>40 mg/L)	31.7 (20.3–45.0)	58.3 (40.8–74.5)	15.9 (6.6–30.1)	33.6 (25.8–42.0)	27.7 (15.6–42.6)
Urinary tract pathologies	29.3 (18.1–42.7)	75.0 (56.6–88.5)	26.2 (13.9–42.0)	39.4 (31.0–48.3)	64.4 (48.8–78.1)

Prevalence categories are classified according to the number of eggs per 10 ml urine.

UTPs were significantly more prevalent (*P* = 0.006) in the adult males (64.4%) than in school-children (39.4%); not surprisingly, given its high prevalence and intensity of infection, as well as its high proportion of heavy infections, Kinyasini school was identified as a hot-spot for UTPs, where the prevalence of the overall positive score (for any UTP) reached 75.0% of school-children in contrast to 27.8% in the remaining schools (*P*<0.0001) (Table 1). Among all the recorded UTPs, bladder pathologies were the most prevalent (24/29 = 82.8% in adult males, and 49/52 = 94.2% in school-children, *P* = 0.10), of which bladder wall irregularities were the commonest (14/24 = 58.3% in adult males, and 38/49 = 77.6% in school-children, *P* = 0.08) (Table 2).

**Table 2:** Number (#) and prevalence (%) of pathologies (UTPs) recorded, in school-children of both sexes (9–15 yr) attending Chaani, Kinyasini and Mwera schools, and adult males (≥16 yr) attending Chaani Health Centre, via ultrasonography in the different tissues of the human urinary tract (bladder, ureters, and renal pelvis) associated with urinary schistosomiasis, Unguja, Zanzibar.

UTPs		Chaani (*N* = 58)		Kinyasini (*N* = 32)		Mwera (*N* = 42)		All schools (*N* = 132)		Health Centre (*N* = 45)	
Tissue		*#*	%	*#*	%	*#*	%	*#*	%	*#*	%
Bladder	Irregular shape	1	1.7	0	0.0	0	0.0	1	0.8	9	20.0
	Wall thickness	9	15.5	6	18.8	2	4.8	17	12.9	5	11.1
	Wall irregularities	9	15.5	21	65.6	8	19.0	38	28.8	14	31.1
	Masses	0	0.0	3	9.4	0	0.0	3	2.3	0	0.0
	Pseudopolyps	0	0.0	0	0.0	0	0.0	0	0.0	0	0.0
	Positive score	16	27.6	24	75.0	9	21.4	49	37.1	24	53.3
Ureters	Pathology in right ureter	2	3.4	1	3.1	0	0.0	3	2.3	4	8.9
	Pathology in left ureter	4	6.9	1	3.1	2	4.8	7	5.3	5	11.1
	Positive score	4	6.9	2	6.3	2	4.8	8	6.1	6	13.3
Renal Pelvis	Right hand side	1	1.7	1	3.1	0	0.0	2	1.5	3	6.7
	Left hand side	3	5.2	0	0	0	0.0	3	2.3	3	6.7
	Positive score	4	6.9	1	3.1	0	0.0	5	3.8	4	8.9
Tissue walls	Calcification	1	1.7	4	12.5	0	0.0	5	3.8	6	13.3
Overall positive score		17	29.3	24	75.0	11	26.2	52	39.4	29	64.4

An overall, binomial score was attributed to the pathological state of individual tissues (positive score), as well as the urinary tract system as a whole (overall positive score), denoting the prevalence of any identifiable pathology according to WHO (1991) standardized methodology.

### Rapid diagnostic tests for urinary schistosomiasis

Comparing to the ‘gold standard’ of microscopy, detecting micro-haematuria by use of Hemastix reagent strips was by far the most reliable and rapid diagnostic tool for the detection of urinary schistosomiasis. The assay was found to be as reliable when used on school-children as when used on adult males (although specificity was lower in the adults) with, respectively, SS = 90.2% and 90.9%, SP = 86.5 and 68.0%, PPV = 79.3 and 71.4%, and NPV = 93.9 and 89.5% (Table 3).

**Table 3:** Comparison between rapid *S. haematobium* diagnostic tests for children (9–15 yr, boys and girls) enrolled in Chaani, Kinyasini, and Mwera schools (*N* = 140), and adults (≥16 yr, males) attending Chaani Health Centre (*N* = 47) on Unguja, Zanzibar: visual blood in urine (macro-haematuria); visual opacity (turbidity); albumin concentration (HemoCue, excreted urine-albumin levels); and urine-reagent strips (Hemastix, micro-haematuria).

Method of diagnosis	Target population	Sensitivity (CI_95_)	Specificity (CI_95_)	PPV (CI_95_)	NPV (CI_95_)
Macro-haematuria (self assessed)	Schools	15.7 (7.0–28.6)	96.6 (90.5–99.3)	72.7 (39.3–94.0)	66.7 (57.8–74.7)
	Health Centre	9.1 (6.8–23.8)	100.0 (86.3–100.0)	100.0 (15.8–100.0)	55.6 (40.0–70.4)
Visual turbidity (bar code chart)	Schools	70.6 (56.2–82.5)	79.8 (69.9–89.6)	66.7 (52.5–78.9)	82.6 (72.9–89.9)
	Health Centre	72.7 (49.8–89.3)	68.0 (46.5–85.1)	66.7 (44.7–84.4)	73.9 (51.6–89.8)
Albuminuria (conc.>40 mg/L)	Schools	74.5 (60.4–85.7)	89.9 (81.7–95.3)	80.9 (66.7–90.9)	86.0 (77.3–92.3)
	Health Centre	31.8 (13.9–54.9)	76.0 (54.9–90.6)	53.8 (25.1–80.8)	55.9 (37.9–72.8)
Micro-haematuria (urine reagent strip)	Schools	90.2 (78.6–96.7)	86.5 (77.6–92.8)	79.3 (66.7–88.8)	93.9 (86.3–98.0)
	Health Centre	90.9 (70.8–98.9)	68.0 (46.5–85.1)	71.4 (51.3–86.8)	89.5 (66.9–98.7)

In all cases parasitological positivity by microscopy (eggs in urine) was the ‘gold standard’.

PPV = positive predictive value; NPV = negative predictive value; CI_95_ = 95% confidence interval.

### Statistical associations—urinary schistosomiasis

In school-children, and after stepwise logistic regression, girls were found less likely to be infected by *S. haematobium* than boys (boys as reference category, OR = 0.4, CI_95_ 0.2–0.9, *P* = 0.032). Additionally, urinary schistosomiasis was found to be associated (albeit not significantly) with self-reported health problems (OR = 2.4, CI_95_ 0.8–7.5, *P* = 0.14), and particularly and significantly with pain on urination (OR = 4.4, CI_95_ 1.7–11.1, *P* = 0.0018). In adult males, and after stepwise logistic regression, urinary schistosomiasis was found to be associated with self-reported pain on urination (OR = 6.3, CI_95_ 1.4–29.6, *P* = 0.019) and urine-flow problems (OR = 3.9, CI_95_ 0.8–19.4, *P* = 0.099).

At the univariate level, urinary schistosomiasis in children was found to be associated with prevalence of UTPs (OR = 11.4, CI_95_ 4.6–27.9, *P*<0.0001), particularly (and after stepwise logistic regression) with having bladder (OR = 7.4, CI_95_ 2.9–18.8, *P*<0.001) and ureteral pathologies (OR = 21.2, CI_95_ 2.0–223.0, *P* = 0.012). Importantly, urinary schistosomiasis in adult males was not found to be statistically significantly associated with exhibiting UTPs in general, nor with having any of the individual pathologies in particular, i.e. bladder, ureteral, or renal pelvis pathologies.

### Statistical associations—elevated urine-albumin concentration (albuminuria)

Elevated urine-albumin concentrations were common in both studied populations (33.6% of school-children and 27.7% of adult males, *P* = 0.48) (Table 1). School-aged boys were more affected than girls, albeit non-significantly (39.7% and 25.5%, respectively, *P* = 0.10). The mean urine-albumin concentration of those found positive for abnormal levels (i.e. >40 mg/L) was 236.2 mg/L in school-children, with affected boys averaging 272.5 mg/L (max. value of 1206 mg/L) and girls averaging 166.5 mg/L (max. value of 408 mg/L), and 96.5 mg/L in adult males (max. value of 288 mg/L).

At the univariate level, a raised urine-albumin level in school-children was found to be highly associated with micro-haematuria (OR = 138.7, *P*<0.0001), suffering UTPs (OR = 7.1, *P*<0.0001), more particularly presenting with bladder (OR = 7.6, *P*<0.0001) and ureteral pathologies (OR = 7.4, *P* = 0.022), and with being parasitologically positive for urinary schistosomiasis (OR = 26.0, *P*<0.0001) (see Table 4, which includes the CI_95_ for the odds ratios). After stepwise logistic regression, school-children diagnosed with micro-haematuria (OR = 76.7, *P*<0.0001) and *S. haematobium* infection (OR = 3.1, *P* = 0.07) were identified more likely to be excreting elevated concentrations of albumin in urine (Table 4).

**Table 4:** Statistical associations of raised urine-albumin levels (>40 mg/L) measured by Albumin-HemoCue, in school-children (9–15 yr, both sexes) and adult males (≥16 yr) (Chaani Health Centre).

Population	Factor	Baseline	Category	Univariate Analysis			Stepwise Logistic Regression		
				OR	CI_95_	*P*-value	OR	CI_95_	*P*-value
Chaani, Kinyasini and Mwera Schools	Micro-haematuria	Negative (≤trace)	Positive (>trace)	138.7	29.6–649.6	<0.0001	76.7	15.5–380.2	<0.0001
	UTPs	Negative	Positive	7.1	3.0–16.7	<0.0001	–	–	–
	Bladder	Negative	Positive	7.6	3.2–17.6	<0.0001	–	–	–
	Ureteral	Negative	Positive	7.4	1.3–39.9	0.022	–	–	–
	Urinary schistosomiasis	Egg-negative	Egg-positive	26.0	10.1–66.5	<0.0001	3.1	0.9–10.5	0.070
Chaani Health Centre	Micro-haematuria	Negative (≤trace)	Positive (>trace)	5.5	1.1–28.6	0.043	–	–	–
	Leukocytes in urine	Negative	Positive	20.6	2.1–202.0	0.009	11.9	1.0–141.0	0.049
	Urine's specific gravity	Lower (<1.02 g/ml)	Higher (≥1.02 g/ml)	4.8	1.1–20.4	0.035	5.7	1.0–32.2	0.050
	Bladder pathologies	Negative	Positive	8.4	1.6–44.5	0.013	4.9	0.8–31.0	0.093

Explanatory variables included urine's chemical/physical properties (pH, specific gravity, protein content, glucose, nitrite and ketone levels, as well as leukocyte presence), urinary tract pathology prevalence (general and specific – bladder, renal pelvis and ureteric pathologies) and urinary schistosomiasis prevalence. Shown are significant associations at univariate level (after accounting for possible intra-correlations in the data), and stepwise (AIC-driven) logistic regression results, enabling identification of the most parsimonius, yet adequate, model for explaining elevated concentration of albuminuria.

At the univariate level, raised urine-albumin level in adult males was found to be associated with having micro-haematuria (OR = 5.5, *P* = 0.043), presence of leukocytes in urine (OR = 20.6, *P* = 0.009), higher urine's specific gravity (≥1.02 g/ml) (OR = 4.8, *P* = 0.035), and presence of bladder pathologies (OR = 8.4, *P* = 0.013). Importantly, albuminuria in adult males was not found to be statistically significantly associated, at the univariate level, with active *S. haematobium* infection. After stepwise logistic regression, the variables that remained in the model explaining excreted albumin in the urine of adult males were presence of leukocytes in urine (OR = 11.9, *P* = 0.049), higher urine's specific gravity (OR = 5.7, *P* = 0.050) and to a lesser extent, presence of bladder pathologies (OR = 4.9, *P* = 0.093).

### Excreted urine-albumin and urinary tract pathologies

Albuminuria was able to identify ultrasonography-positive UTPs more effectively in school- children (SS = 61.5%, SP = 83.8%, PPV = 71.1%, NPV = 77.0%) than in the adult male population surveyed at Chaani Health Centre (SS = 37.9%, SP = 87.5%, PPV = 84.6%, NPV = 43.8%). Additionally, the diagnostic performance of this test increased somewhat when the diagnostic target was changed to specific pathology of the bladder instead of general UTPs (school-children: SS = 63.3%, SP = 83.1%, PPV = 68.9%, NPV = 79.3%; adults: SS = 45.8%, SP = 90.5%, PPV = 84.6%, NPV = 59.4%) (see Table 5 for confidence intervals around these diagnostic point estimates, and see Table S1 for diagnostic performance of albuminuria at school level).

**Table 5:** Diagnostic performance of albuminuria (>40 mg of albumin per litre of urine), measured by Albumin-HemoCue photometer, for identifying general urinary tract pathologies (UTPs) or more particularly bladder pathologies in children (9–15 yr, boys and girls) enrolled in Chaani, Kinyasini, and Mwera schools (*N* = 132), and adults (≥16 yr, males) attending Chaani Health Centre (*N* = 47) on Unguja, Zanzibar.

Diagnostic target	Diagnostic parameter	Schools	Health Centre
UTPs	Sensitivity (%/CI_95_)	61.5 (47.0–74.7)	37.9 (20.7–57.7)
	Specificity (%/CI_95_)	83.8 (73.8–91.1)	87.5 (61.7–98.5)
	PPV (%/CI_95_)	71.1 (55.7–83.6)	84.6 (54.6–98.1)
	NPV (%/CI_95_)	77.0 (66.8–85.4)	43.8 (26.4–62.3)
Bladder pathologies	Sensitivity (%/CI_95_)	63.3 (48.3–76.6)	45.8 (25.6–67.2)
	Specificity (%/CI_95_)	83.1 (73.3–90.5)	90.5 (69.6–98.8)
	PPV (%/CI_95_)	68.9 (53.4–81.8)	84.6 (54.6–98.1)
	NPV (%/CI_95_)	79.3 (69.3–87.3)	59.4 (40.6–76.3)

Ultrasound identification of pathologies was the ‘gold standard’. Refer to table S1 for further information on diagnostic performances at the school level.

PPV = positive predictive value; NPV = negative predictive value; CI_95_ = 95% confidence interval.

## Discussion

Urinary schistosomiasis in school-children was found to be significantly associated with gender, with boys being twice as likely to be infected as girls, in agreement with previous observations in Zanzibar [32]. Although water contact behaviour, a potential confounder, was not taken into consideration regarding the association between urinary schistosomiasis and gender, our results concur with those of other studies [31]. Feeling unwell, in particular reporting complaints regarding pain on urination, was also found to be positively associated with urinary schistosomiasis in school-children. The lack of stronger association concerning self-reported health problems reflects the large number of asymptomatic infections in this younger age-class [33]. School-children infected by *S. haematobium* were also more likely to have UTPs, particularly bladder and ureteral pathologies.

In the adult male population surveyed at Chaani Health Centre, urinary schistosomiasis was found to be associated with self-reported pain on urination, in particular around the bladder, and self-reported urine flow problems, illustrating the damage caused by egg expulsion through the urinary tract, even though no statistically significant association was found between active *S. haematobium* infection and UTPs.

In endemic, untreated populations, the prevalence and intensity of schistosomiasis is usually higher in children than in adults, giving rise to typically convex age-infection profiles [34]. In this study, however, the adult male population surveyed at the Chaani Health Centre had a higher prevalence of urinary schistosomiasis than the school-children (overall infection prevalence of 46.8% vs. 36.4%, and heavy infection prevalence of 19.2% vs. 15.7%, respectively) but none of these differences were statistically significant. However, recruitment bias in the health centre must be considered before comparing this study's results with those of [34], as adult women were not included, and the males recruited, although randomly selected, constitute a small sample size and may have been seeking medical assistance for some of the complaints investigated in this study.

Importantly, Kinyasini was identified as a high infection school (overall infection prevalence of 72.2%), where 41.7% of the school-children were heavily infected with *S. haematobium*, even with ongoing treatment (therapeutic coverage at school = 80%), and access to potable water (94%). Kinyasini also had a significantly higher prevalence of UTPs than Chaani and Mwera schools. The infection status of this school (which was statistically significantly higher than that of the two remaining schools considered together) is believed to be the consequence of behavioural risk factors particularly prevalent in this population, such as extremely frequent contact with contaminated waters by the children [35], and its close geographical proximity to highly contaminated water sources [32].

### Urinary tract pathologies in Zanzibar

The prevalence of UTPs was significantly higher in adult males (64.4%) compared to that in school-children (39.4%), reflecting the chronic nature of these sequelae. Additionally, this could be the result of ongoing chemotherapy campaigns targeting school-children, not only impacting infection prevalence and intensity, but also diminishing chronic disease progression [36]. Interestingly, in 1966 the prevalence of UTPs in Ungujan school-children was reported to be 25.3% (*N* = 363) [37], which was significantly lower than that reported here (*P* = 0.018), although the first survey was conducted on a different village using a different methodology to identify UTPs.

### Micro-haematuria and albuminuria are effective indicators of infection

The use of urine filtration for detection of *S. haematobium* eggs as the diagnostic methodology in large-scale surveys has been criticised as being not as cost-effective as alternative diagnostic methods, particular when it comes to identifying high-risk schools, or individuals with high worm burdens in low-risk schools ([38] in particular discusses the cost-effectiveness of self-reported blood in urine). The low cost (although not as low as that of microscopy), ease of use, rapidity, and strong diagnostic power of the urine-reagent strips (e.g. Hemastix) have been previously established, demonstrating this to be the best rapid-diagnostic test for *S. haematobium* infections [2],[11],[12]; a result confirmed in the present study.

Additionally, assays to detect albuminuria (>40 mg/L) were confirmed as an effective rapid diagnostic tool for urinary schistosomiasis in school-children (SS = 74.5%, SP = 89.9%, PPV = 80.9%, NPV = 86.0%), as previously reported by [7], who estimated somewhat higher values in a cohort of 305 Ungujan school-children. This assay performed less satisfactorily in the 47 adult males surveyed (Table 3).

### Models of excreted urine-albumin in school-children and adult males

In univariate analyses, albuminuria, as defined in [7], was found to be statistically and significantly associated with UTPs in both school-children and adult males, though statistical significance was not retained in the multivariate analyses. Additionally, whereas albuminuria is efficient at identifying active *S. haematobium* infection in school-children ([7] and above), its diagnostic power was largely decreased when the test was performed on adult males.

A raised urine-albumin level was found to be associated with the presence of micro-haematuria in school-children, whereas in adults a raised urine-albumin level was found to be significantly associated with the presence of leukocytes and higher urine's specific gravity (>1.02 g/ml). Although the presence of bladder pathologies was retained in the final, multivariate model of albuminuria for adult males, this variable was not statistically significant possibly because of lack of power due to small sample size. The latter observations (together with the absence of micro-haematuria as a significant covariate in the final statistical model for adult males) are possibly indicative of scarring in the urinary tract of adult males, whereas school-children appear to have raised albumin concentrations in their urine due to active egg expulsion and consequent haematuria.

Albuminuria can be indicative of kidney disease, hyperglycaemia, and hypertension [39], with micro-albuminuria defined as levels of albumin in urine greater than 30 mg/L but lower than 300 mg/L in 24 hours, and gross albuminuria as levels exceeding 300 mg/L. We did not distinguish between these two, and it would be interesting to ascertain in larger cohorts of adults whether gross albuminuria correlates positively and significantly with chronic and severe UTPs due to *S. haematobium*. Another important observation was the increase in the diagnostic power of urine-albumin assays when identifying bladder pathologies in particular, and not just UTPs in general. This fact can be explained if we consider bladder pathologies, chiefly bladder wall lesions, as the major source of albumin-rich blood-serum in urine.

**Figure 1:**
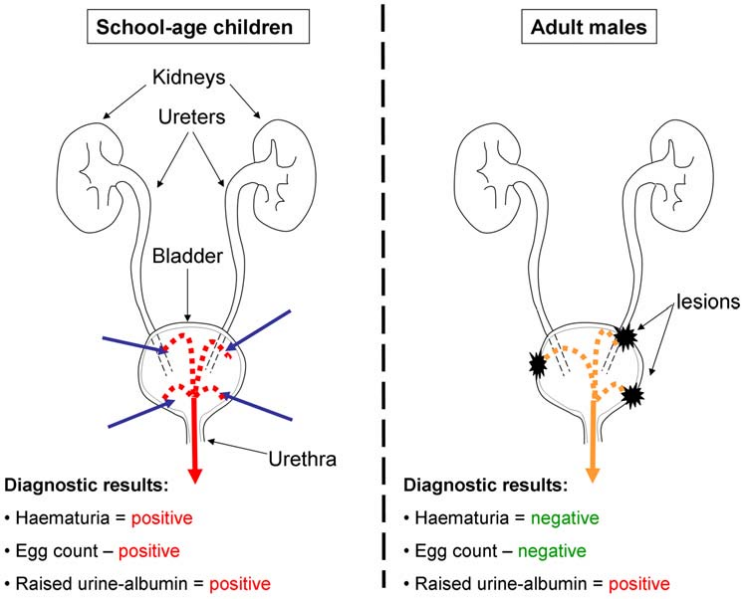
Proposed aetiology of raised urine-albumin levels in school-children and adults. In school-children (left), worms are likely located in the vesical plexus. As egg production ensues, eggs pass from the lumen of blood vessels into adjacent tissues, and many penetrate the bladder mucosa being shed into the urine (blue arrows). This causes tissue damage and subsequent haemorrhage (red broken lines and arrow) identifiable using urine-reagent strips (micro-haematuria) and/or visually (macro-haematuria). The presence of blood will increase the levels of albumin in urine (albuminuria). In adults (right), chronic manifestations of past and present *S. haematobium* are common, particularly bladder wall lesions. Through these lesions, blood serum and white blood cells (leukocytes), as well as other components of the human immune system, infiltrate the bladder (orange broken lines and arrow) from the several capillary vessels, responding to scarring and attempting to reduce risk of subsequent urological infections through the already damaged epithelial barrier. Blood serum is rich in albumin, and its leaching into the urine will increase protein concentration; importantly, this will occur without the direct influence of *S. haematobium* egg excretion and in the absence of haematuria.

In view of the above, and for populations living in urinary schistosomiasis endemic areas of Africa, we propose a dual mechanism for explaining raised albumin concentrations in the urine of school-children, associated with ongoing perforation of the urinary tract by *S. haematobium* eggs and traces of blood in urine, and adult males, associated with bladder wall pathologies (Fig. 1). Importantly, as control campaigns progress and treatment becomes widely available, the prevalence of infection will decrease, rendering albuminuria more effective, in the absence of the confounding effect of infection-related micro-haematuria, at detecting UTPs in the target population (i.e. the school-aged children).

### Costings and logistics

Targeting children at schools is thought to be highly effective in the control of schistosomiasis, not only because school-children are at higher-risk of infection [40], but also because treatment of this age-group offers great reversibility of disease morbidity and its manifestations [33]. Current monitoring of control campaigns measures effectiveness according to reductions in infection prevalence and intensity, as well as reductions in anaemia, haematuria, improvements in nutrition status or school-attendance [41]. In this paper, we advocate that prevalence of UTPs should also be considered, as they are a major consequence of long-term and continuous exposure to *S. haematobium* (see also [42]).

As the fight against schistosomiasis progresses, today's target populations, the school-children, will be tomorrow's adults. The impact of past and present control initiatives needs to be assessed not only in school-children but also in adults to help assess the progression of temporal and age-specific trends in infection and morbidity as chemotherapy-based control programmes advance. In adults, infection prevalence and intensities are often lower than in children at endemic equilibrium [34]. Successful control campaigns will minimise the prevalence and incidence of acute disease manifestations (e.g. haematuria). Nonetheless, chronic pathologies, particularly those of the urinary tract, will still be present, as their improvement, even in the face of treatment, can only be tangible in the long-term, emphasizing the importance of monitoring such pathologies. Portable ultrasonography, however, is limited as a field-based methodology; new protocols for the identification of UTPs should therefore be investigated. At the most basic level (i.e. consumables), the combined cost of testing a urine sample for the presence of blood and albuminuria (£0.23 per individual Hemastix strip+£1.75 for individual HaemoCue cuvettte), compares less favourably than the cost of performing parasitological microscopy (£0.08 per test) and ultrasonography (£0.10 per examination). Nevertheless, we must consider that the latter techniques demand more expensive equipment, i.e. microscope and portable ultrasound machine, as well as trained technicians.

## Conclusions

In this paper, albuminuria (defined as albumin concentration in urine >40 mg/L) has been found to be a putative indicator of urinary tract pathologies, and it should be considered as a future rapid morbidity assessment tool to be used in conjunction with urine-reagent strips (e.g. Hemastix) for the screening and monitoring of urinary schistosomiasis, as well as associated chronic morbidity, before and during interventions, especially where ultrasonography may not be possible.

## Supporting Information

Table S1 Diagnostic potential of albuminuria at the school level.
(0.04 MB DOC)

Alternative Language Abstract S1 Translation of the abstract into Portuguese by JCSF.
(0.03 MB DOC)

Alternative Language Abstract S2 Translation of the abstract into Spanish by MGB.
(0.03 MB DOC)
