# Supplementary material for: Measuring Morbidity Associated with Urinary Schistosomiasis: Assessing Levels of Excreted Urine Albumin and Urinary Tract Pathologies
Source: PLoS Negl Trop Dis. 2009 Oct 6;3(10):e526. doi: 10.1371/journal.pntd.0000526 (PMC2752803; doi:10.1371/journal.pntd.0000526)
Supplement: Table S1 — Diagnostic potential of albuminuria at the school level. (0.04 MB DOC) [file pntd.0000526.s001.doc]

**SUPPLEMENTARY MATERIAL**

**Table S1.** Diagnostic performance of albuminuria (>40mg of albumin per litre of urine), measured by Albumin-HemoCue® photometer, for identifying general urinary tract pathologies (UTPs) or more particularly bladder pathologies in children (9-15 yr, boys and girls) enrolled in Chaani (*N*=58), Kinyasini (*N*=32) and Mwera (*N*=42) schools on Unguja, Zanzibar. Ultrasound identification of pathologies was the ‘gold standard’

| Diagnostic target | Diagnostic parameter | Chaani | Kinyasini | Mwera |
| --- | --- | --- | --- | --- |
|  |  |  |  |  |
| UTPs | Sensitivity (%/CI_95_) | 70.6%  (44.0 - 89.7%) | 75.0%  (53.3 - 90.2%) | 18.2%  (2.3 - 51.8%) |
|  | Specificity (%/CI_95_) | 85.4%  (70.8 - 94.4%) | 75.0%  (34.9 - 96.8%) | 83.9%  (66.3 - 94.6%) |
|  | PPV (%/CI_95_) | 66.7%  (41.0 - 86.7%) | 90.0%  (68.3 - 98.8%) | 28.6%  (3.7 - 71.0%) |
|  | NPV (%/CI_95_) | 87.5%  (73.2 - 95.8%) | 50.0%  (21.1 - 78.9%) | 74.3%  (56.7 - 87.5%) |
|  |  |  |  |  |
| Bladder pathologies | Sensitivity (%/CI_95_) | 68.8%  (41.3 - 89.0%) | 75.0%  (53.3 - 90.2%) | 22.2%  (2.8 - 60.0%) |
|  | Specificity (%/CI_95_) | 83.3%  (68.6 - 93.0%) | 75.0%  (34.9 - 96.8%) | 84.8%  (68.1 - 94.9%) |
|  | PPV (%/CI_95_) | 61.1%  (35.8 - 82.7%) | 90.0%  (68.3 - 98.8%) | 28.6%  (3.7 - 71.0%) |
|  | NPV (%/CI_95_) | 87.5%  (73.2 - 95.8%) | 50.0%  (21.1 - 78.9%) | 80.0%  (63.1 - 91.6%) |
|  |  |  |  |  |

PPV = positive predictive value; NPV = negative predictive value; CI_95_ = 95% confidence interval.
