# Supplementary material for: Measuring Morbidity Associated with Urinary Schistosomiasis: Assessing Levels of Excreted Urine Albumin and Urinary Tract Pathologies
Source: PLoS Negl Trop Dis. 2009 Oct 6;3(10):e526. doi: 10.1371/journal.pntd.0000526 (PMC2752803; doi:10.1371/journal.pntd.0000526)
Supplement: Alternative Language Abstract S1 — Translation of the abstract into Portuguese by JCSF. (0.03 MB DOC) [file pntd.0000526.s002.doc]

**Medindo a morbidade associada com a esquistossomose urinária: Níveis de albumina na urina e patologias do tracto urinário**

**José C. Sousa-Figueiredo^1^*, María-Gloria Basáñez^2^, I. Simba Khamis^3^, Amadou Garba^4^, David Rollinson^1^, J. Russell Stothard^1^**

**1** Wolfson Wellcome Biomedical Laboratories, Department of Zoology, Natural History Museum, Cromwell Road, London SW7 5BD, UK, **2** Department of Infectious Disease Epidemiology, Imperial College London, Norfolk Place, London W2 1PG, UK, **3** Helminth Control Laboratory Unguja, Helminth Control Programme, Zanzibar, Tanzania, 4 Réseau International Schistosomoses Environnement Amenagements et Lutte (RISEAL-Niger), 333, Avenue des Zarmakoye, BP.13724, Niamey, Niger

* E-mail: j.figueiredo@nhm.ac.uk

Título curto: **Albuminuria & Urinary tract pathology in Zanzibar**

**Sumário**

A esquistossomose urinária é responsavel por uma variedade de condições debilitantes; acima de todas, talvez, estão as patologias do tracto urinário. Embora aparelhos de ultra-som portáteis possam ser utilizados para detectar estas patologias visualmente, existe uma necessidade de ferramentas de avaliação rápida de morbidade (doravante referidas como RaMA) que possam ser implantadas no campo durante a execução, acompanhamento e avaliação dos programas de controle. Nós, portanto, tentamos com este artigo determinar as associações entre albuminúria, medida usando um fotómetro de HemoCue, e patologias do tracto urinário, detectadas visualmente por ultrasonografia, em crianças e adultos de uma zona endémica da esquistossomose urinária em Zanzibar.

***Metodologia/ resultados principais:*** Em um levantamento de 140 crianças de idade escolar (dos 9 a 15 anos) de ambos os sexos e 47 homens adultos (≥16 anos de idade) as prevalências da esquistossomose urinária (ovo-patente) eram 36.4% (CI_95_ 28.5-45.0%) e 46.8% (CI_95_ 32.1-61.9%) (*P* = 0.14), e de patologias do tracto urinário 39.4% (CI_95_ 31.0-48.3%) e 64.4% (CI_95_ 48.8-78.1%) (*P* = 0.006), respectivamente. Em crianças de idade escolar, concentrações elevantadas de albumina de urina (>40 mg/L) estavam associados, embora não-significativamente, com prevalência de infecção (OR = 3.1, *P* = 0.070), mas mais especificamente e significativamente com a prevalência de micro-hematúria (OR = 76.7, *P* < 0.0001). Nos adultos, a excreção elevada de albumina na urina estava associada com patologias do tracto urinário, particularmente as lesões da parede da bexiga (OR = 8.4, P = 0.013). Albuminúria revelou um desempenho diagnóstico promissor, especialmente em crianças de idade escolar com sensibilidade de 63.3% e especificidade de 83.1% na detecção de patologias do tracto urinário inferior, ou seja, as lesões de parede da bexiga (ultrasonografia como ‘padrão-ouro’).

***Conclusão/significância:*** Este estudo indica que albuminuria poderia ser utilizada como uma ferramenta de RaMA para acompanhamento da prevalência de patologias do tracto urinário durante programas de controlo de esquistossomose urinária, bem como uma ferramenta para selecionar aqueles com as lesões mais crónicas na parede da bexiga sem recorrer à ultrasonografia.
