# Supplementary material for: Measuring Morbidity Associated with Urinary Schistosomiasis: Assessing Levels of Excreted Urine Albumin and Urinary Tract Pathologies
Source: PLoS Negl Trop Dis. 2009 Oct 6;3(10):e526. doi: 10.1371/journal.pntd.0000526 (PMC2752803; doi:10.1371/journal.pntd.0000526)
Supplement: Alternative Language Abstract S2 — Translation of the abstract into Spanish by MGB. (0.03 MB DOC) [file pntd.0000526.s003.doc]

**Cuantificando la morbilidad asociada con la esquistosomiasis urinaria: niveles de albuminuria y patologías del tracto urinario**

**José C. Sousa-Figueiredo^1^*, María-Gloria Basáñez^2^, I. Simba Khamis^3^, Amadou Garba^4^, David Rollinson^1^, J. Russell Stothard^1^**

**1** Wolfson Wellcome Biomedical Laboratories, Department of Zoology, Natural History Museum, Cromwell Road, London SW7 5BD, UK, **2** Department of Infectious Disease Epidemiology, Imperial College London, Norfolk Place, London W2 1PG, UK, **3** Helminth Control Laboratory Unguja, Helminth Control Programme, Zanzibar, Tanzania, 4 Réseau International Schistosomoses Environnement Amenagements et Lutte (RISEAL-Niger), 333, Avenue des Zarmakoye, BP.13724, Niamey, Niger

* E-mail: j.figueiredo@nhm.ac.uk

Título Corto: **Albuminuria y patología del tracto urinario en Zanzíbar**

**Resumen**

***Antecedentes:*** La esquistosomiasis urinaria es responsable por una variedad de condiciones debilitantes, entre las cuales las patologías del tracto urinario (PTU) son posiblemente las más importantes. Si bien la ultrasonografía puede ser utilizada para detección visual de las PTU, existe aún la necesidad de desarrollar herramientas para la evaluación rápida de la morbilidad (de aquí en adelante referida como RaMA) que puedan emplearse en el campo durante la implementación, el monitoreo, y la evaluación de programas de control. Nuestro objetivo fue el de determinar asociaciones entre albúmina excretada en la orina (albuminuria, medida mediante fotómetro HemoCue) y PTU (detectadas por ultrasonografía) en niños y adultos en un área endémica para la esquistosomiasis urinaria en Zanzíbar.

***Metodología / Hallazgos Principales:*** Realizamos un estudio de 140 niños escolarizados pertenecientes a ambos sexos (de edades comprendidas entre los 9 y los 15 años) y 47 adultos del sexo masculino (de edades superiores a los 16 años) en la isla de Unguja. La prevalencia de esquistosomiasis (detectada por la presencia de huevecillos en la orina) fue de 36.4% (IC_95_ 28.5-45.0%) en los niños, y de 46.8% (IC_95_ 32.1-61.9%) en los adultos (*P* = 0.14). La prevalencia correspondiente de PTU fue de 39.4% (IC_95_ 31.0-48.3%) y 64.4% (IC_95_ 48.8-78.1%) (*P* = 0.006), respectivamente. En los niños, concentraciones de albuminuria por encima de los 40 mg por litro (>40 mg/L) se hallaron positivamente asociadas con la prevalencia de infección por *Schistosoma haematobium* (OR = 3.1, *P* = 0.070), pero dicha asociación fue más específica y estadísticamente significativa con la prevalencia de micro-hematuria (OR = 76.7, *P* < 0.0001). En los adultos, un nivel elevado de albuminuria estuvo asociado con PTU, particularmente con lesiones de la pared de la vejiga urinaria (OR = 8.4, *P* = 0.013). En comparación con la ultrasonografía (como el estándar de oro), la albuminuria fue capaz de diagnosticar patologías del tracto urinario inferior, especialmente en los niños de edad escolar con una sensibilidad del 63.3%, y una especificidad del 83.1%.

***Conclusiones / Significado*:** Este estudio indica que el uso de pruebas de albuminuria podría contribuir a la evaluación rápida de la morbilidad (RaMA) en la esquistosomiasis urinaria, tanto para el monitoreo de la prevalencia de las PTU durante programas de control, como para la identificación de aquellas personas con lesiones crónicas de la pared de la vejiga obviando la necesidad de ultrasonografía.
